# Regulation of Viral Replication, Apoptosis and Pro-Inflammatory Responses by 17-AAG during Chikungunya Virus Infection in Macrophages

**DOI:** 10.3390/v9010003

**Published:** 2017-01-06

**Authors:** Tapas K. Nayak, Prabhudutta Mamidi, Abhishek Kumar, Laishram Pradeep K. Singh, Subhransu S. Sahoo, Soma Chattopadhyay, Subhasis Chattopadhyay

**Affiliations:** 1 School of Biological Sciences, National Institute of Science Education & Research, Bhubaneswar, HBNI, Jatni, Khurda, Odisha 752050, India; tapas.sbsniser@gmail.com (T.K.N.); laishrampk@gmail.com (L.P.K.S.); subhransusahoo87@gmail.com (S.S.S.); 2 Infectious Disease Biology, Institute of Life Sciences, (Autonomous Institute of Department of Biotechnology, Government of India), Nalco Square, Bhubaneswar, Odisha 751023, India; rinku.prabhu@gmail.com (P.M.); abhishekbt13@gmail.com (A.K.)

**Keywords:** Chikungunya virus, Alphavirus, macrophage, MHC, TNF, HSP90, apoptosis, 17-AAG

## Abstract

Chikungunya virus (CHIKV) infection has re-emerged as a major public health concern due to its recent worldwide epidemics and lack of control measures. Although CHIKV is known to infect macrophages, regulation of CHIKV replication, apoptosis and immune responses towards macrophages are not well understood. Accordingly, the Raw264.7 cells, a mouse macrophage cell line, were infected with CHIKV and viral replication as well as new viral progeny release was assessed by flow cytometry and plaque assay, respectively. Moreover, host immune modulation and apoptosis were studied through flow cytometry, Western blot and ELISA. Our current findings suggest that expression of CHIKV proteins were maximum at 8 hpi and the release of new viral progenies were remarkably increased around 12 hpi. The induction of Annexin V binding, cleaved caspase-3, cleaved caspase-9 and cleaved caspase-8 in CHIKV infected macrophages suggests activation of apoptosis through both intrinsic and extrinsic pathways. The pro-inflammatory mediators (TNF and IL-6) MHC-I/II and B7.2 (CD86) were also up-regulated during infection over time. Further, 17-AAG, a potential HSP90 inhibitor, was found to regulate CHIKV infection, apoptosis and pro-inflammatory cytokine/chemokine productions of host macrophages significantly. Hence, the present findings might bring new insight into the therapeutic implication in CHIKV disease biology.

## 1. Introduction

Chikungunya virus (CHIKV), a mosquito borne re-emerging Alphavirus, belonging to the Togaviridae family, is endemic mainly in Africa, India, China and many other parts of Asia [1,2,3,4]. It was first isolated from Tanzania (formerly Tanganyika), Africa in 1952 during an epidemic of dengue-like illness [5]. Chikungunya fever (CHIKF) is often characterized by sudden appearance of high fever, headache, nausea, vomiting, rashes over skin [6] and followed by polyarthralgia, myalgia [7,8] and gastrointestinal complaints [9]. In some cases, complications such as myopericarditis [10], retrobulbar neuritis [11,12], nephritis [13], myocarditis, pericarditis [14], meningoencephalitis and death, have also been reported in CHIKV infection [9,15,16]. CHIKV is mainly transmitted to the vertebrate host by *Aedes* mosquitoes and maintained in two distinct transmission cycles: urban cycle between human and mosquitoes and sylvatic cycle within forest dwelling mosquitoes and non-human primates [17]. It is an enveloped virus, containing 11.8 kb long single stranded positive sense RNA genome with two open reading frames (ORF). The 5′ ORF codes for non-structural proteins, nsP1-4, mainly involved in viral replication and 3′ ORF codes for three major structural proteins, capsid, E1 and E2 [18].

Like other viral infections, following inoculation, CHIKV induces strong and quick type I interferon (IFN) response. Initially, it has been suggested that wild type adult mice but not the neonates are resistant to CHIKV infection and in neonates’ disease severity is age dependent. Adult mice with IFN-α/βR^+/−^ or IFN-α/βR^−/−^ develop a mild or severe CHIKV infection respectively [19]. Moreover, it has been reported that CHIKV nsP2 can suppress anti-viral pathway by inhibiting IFN-α/β receptor signaling [20]. CHIKF is also associated with increased level of pro-inflammatory cytokines IL-1β, IL-6, IL-12, TNF, IFN-γ [21,22,23,24], GM-CSF [25,26], chemokines IL-8, and MCP-1 [27,28,29], as well as decreased level of pro-inflammatory chemokine RANTES [30].

CHIKV targets a wide range of immune and non-immune cells for its replication, propagation and dissemination. Among them, antigen presenting cells (APCs) such as macrophages/monocytes are known to play important roles towards modulating adaptive immune response against pathogens [31,32,33,34,35]. Among blood leukocytes, monocytes are known to be the major host cells for acute CHIKV infection in humans [36]. Previous studies have shown that macrophages could also be infected with CHIKV, both in vivo [37] and in vitro [22]. In both mouse and macaque models, it has been found that CHIKV induces predominant infiltration of monocytes, macrophages and NK cells with the production of MCP-1, TNF and IFN-γ at the site of inoculation, suggesting a strong immune activation [38]. This productive infection of CHIKV in macrophages could be associated with arthritis, tenosynovitis and myositis [38] despite a robust immune activation [39].

Recent reports have shown that viral infection often induces expression of various intracellular stress related proteins. The heat shock proteins (HSP) are molecular chaperones which bind and stabilize misfolded or unfolded polypeptides to ensure their proper folding and assembly with other polypeptides to decipher the normal protein function [40,41,42,43]. Induction of HSPs has been reported in both RNA and DNA viral infections, however, the type of HSP involved in a viral infection depends on the kind of virus and the type of host cells associated to the infection [44,45]. Recent studies have shown the functional requirement of HSP90 for Human cytomegalo virus (HCMV), Hepatitis C virus (HCV), Herpes Simplex virus-1 (HSV-1), Human Immunodeficiency virus-1 (HIV-1), Hepatitis E virus (HEV), Epstein Barr virus (EBV), Vaccinia virus and rotavirus infections [46,47,48,49,50,51,52,53]. The protein expression of HSP90 usually does not change during viral infection in macrophages [54], however macrophage immune responses for antigen presentation, phagocytosis and inflammatory responses are affected by functional HSP90.

Recently, it has been reported that Geldanamycin (GA) [55] and two other newly synthesized HSP90 inhibitory drugs (HS-10 and SNX-2112) [56] can regulate CHIKV infection both in vitro and in vivo. However, a functional role of HSP90 in CHIKV replication and associated immune modulation in macrophages during infection remains obscured. Accordingly, we have tested whether 17-AAG, a HSP90 functional inhibitor and a proposed therapeutic drug [57], has any regulatory role in CHIKV infection, apoptosis and the altered host immune response in macrophages.

## 2. Materials and Methods

### 2.1. Cells and Viruses

The Indian outbreak strain of CHIKV, DRDE-06 (accession no. EF210157.2), CHIKV prototype strain S 27 (accession no. AF369024.2) and Vero cells (African green monkey kidney epithelial cell line) were kind gifts from Dr. Manmohan Parida, Defence Research & Development Establishment (DRDE), Gwalior, India. The mouse monocyte/macrophage cell line, Raw264.7 (ATCC^®^ TIB-71™) was maintained in RPMI-1640 (HiGlutaXL™ RPMI-1640) supplemented with 2.0 mM l-glutamine, Penicillin 100 U/mL, Streptomycin 0.1 mg/mL (Himedia Laboratories Pvt. Ltd., Mumbai, India), 10% Fetal bovine serum (FBS; PAN Biotech, Aidenbach Germany) at 37 °C under a humidified incubator with 5% CO_2_. Vero cells were maintained in Dulbecco’s modified Eagle’s medium (DMEM; PAN Biotech) supplemented with 5% FBS, Gentamycin (Sigma-Aldrich, St. Louis, MO, USA). The enzyme free cell dissociation reagent (ZymeFree™; Himedia Laboratories, Pvt. Ltd., Mumbai, India) was used for subculturing the cells.

### 2.2. Antibodies and Reagents

The mouse anti-CHIKV-nsP2 monoclonal antibody, used in this study was developed by us [58]. The anti-CHIKV-E2 monoclonal antibody was a kind gift from Dr. Manmohan Parida, DRDE, Gwalior, India. HRP linked secondary antibodies, H-2k^d^ PE, I-A/I-E PE, isotype PE, isotype APC and HSP90 antibodies were purchased from BD Biosciences (San Jose, CA, USA). CD86 APC and CD80 APC were purchased from eBiosciences (San Diego, CA, USA). The monoclocal antibodies for cleaved caspase-3 (Asp175), cleaved caspase-8 (Asp387) and caspase-9 (C9) were purchased from cell signaling technology (Danvers, MA, USA). The anti-mouse Alexa Fluor 488 was purchased from Invitrogen (Carlsbad, CA, USA). Mouse IgG1 isotype control, GAPDH and β-actin were purchased from Abgenex India Pvt. Ltd. (Bhubaneswar, India). Saponin, Anisomycin and Bovine serum albumin fraction V were purchased from Sigma-Aldrich. 17-Allylaminogeldanamycin (17-AAG) and Z-VAD-FMK were purchased from Merck Millipore (Billerica, MA, USA).

### 2.3. MTT Assay 

MTT assay was performed to assess cytotoxicity of 17-AAG and Z-VAD-FMK using EZcount™ MTT cell assay kit (Himedia Laboratories Pvt. Ltd., Mumbai, India) according to the manufacturer’s instructions. Briefly, the Raw264.7 cells were seeded in 96 well plate at a density of 5 × 10^3^ cells per well before drug treatment. The cells were then washed in 1× PBS and incubated with different concentrations of drugs in triplicate. The DMSO was taken as solvent control. After 24 h of treatment, the cells were incubated with the MTT reagent to a final concentration of 10% of the total volume. Then, the cells were incubated for 2 h in the incubator for the formation of visible crystals. Later, the media was removed carefully and 100 µL of solubilization solution was added per well followed by incubation for 15 min at room temperature (RT). The absorbance of the solution was taken at 550 nm by Microplate Reader (Bio-Rad, Hercules, CA, USA). Next, the percent viable cells were calculated in comparison to the control cells of the same plate in triplicate.

### 2.4. CHIKV Infection in Macrophage

The Raw264.7 cells of low passage number were seeded in six well plate before 18–20 h of infection with 60%–70% confluency. The cells were infected with DRDE-06 strain of CHIKV with different multiplicity of infection (MOI) as described earlier [59]. Briefly, the cells were washed in 1× PBS and the virus was added over confluent monolayer for 2 h in the incubator with manual shaking at an interval of 15 min. Then, the virus inoculum was washed in 1× PBS and the cells were maintained at 37 °C in complete RPMI-1640 media. The infected cells and the supernatants were collected at different time points and subjected to further processing according to the assay. Further, the CHIKV infected and the mock cells were examined under microscope (20× magnification) and pictures were taken at different hours post infection (hpi) to observe the cytopathic effect (CPE).

Vero cells with at least 90% confluency were seeded in 35 mm^2^ cell culture dishes in complete media. Next day, the cells were infected with different strains of CHIKV as described previously [59]. The cells and supernatants were collected at different hpi and processed according to the assay.

For 17-AAG or Z-VAD-FMK treatment, the infection was carried out for 2 h in the presence of solvent control, DMSO or the drug. The cells were washed thoroughly with 1× PBS after 2 h and cultured in serum free media containing the drug for 3 h. Then, serum was added to the cells and maintained in the incubator until harvesting [55].

### 2.5. Plaque Assay

For the determination of the virus titer, plaque assay was performed on the Vero cells as described previously [59]. Briefly, after infecting the Vero cells using cell culture supernatants collected from CHIKV infected Raw cells, the cells were overlaid with complete DMEM containing methyl cellulose and maintained in 37 °C incubator. After development of the visible plaques (4–5 days), the cells were fixed in formaldehyde at room temperature, washed gently in distilled water and stained with crystal violet. Then, the numbers of plaques were counted manually under white light.

### 2.6. Flow Cytometry (FC)

Flow cytometric assay was performed as mentioned elsewhere [60]. In brief, mock and CHIKV infected Raw264.7 cells were harvested by scraping, fixed in 4% paraformaldehyde for 10 min at RT and were washed twice in ice cold 1× PBS to remove excess paraformaldehyde. Then, the cells were resuspended in FACS buffer (1× PBS, 1% BSA, 0.01% NaN_3_) and stored at 4 °C until staining. For intra cellular staining (ICS) of CHIKV antigens, the cells were permeabilized in permeabilization buffer (1× PBS + 0.5% BSA + 0.1% Saponin + 0.01% NaN_3_) followed by blocking in 1% BSA (in permeabilization buffer) for 30 min at RT. Then, the cells were washed with permeabilization buffer, incubated with anti-CHIKV-nsP2 or E2 antibodies [58], washed two times in permeabilization buffer followed by incubation in Alexa Fluor^®^ 488 conjugated chicken anti-mouse IgG (H + L) secondary antibody. Both the primary and secondary antibodies were diluted in permeabilization buffer. The mouse IgG was taken as an isotype control during ICS. For the surface staining (CS), the fixed cells were washed with ice cold FACS buffer and then, fluorophore conjugated antibodies against different immune markers were added (diluted in FACS buffer). The mock and CHIKV infected cells were incubated with antibodies for 30 min on ice and washed twice with ice cold FACS buffer to remove un-bound antibodies. The fluorophore compatible isotype control antibodies were used as an isotype control for CS. The FcR blocking reagent (Miltenyi biotec, Bergisch Gladbach, Germany) was used at a dilution of 1:20 prior to the primary antibody incubation to prevent non-specific binding of antibodies to the Fc receptors on macrophages. Then, the cells were acquired by the BD FACS Calibur^TM^ flow cytometer (BD Biosciences) and analyzed by the CellQuest Pro software (BD Biosciences). A total of approximately ten thousand cells were acquired per sample.

### 2.7. Sandwich ELISA for Cytokine Analysis

Cytokine production by the macrophages was analyzed from the cell culture supernatants as mentioned in methods by the BD OptEIA™ sandwich ELISA kit (BD Biosciences) according to the manufacturer’s instructions. The cytokine concentrations in the test samples were calculated in comparison with the corresponding standard curve that was constructed using different concentrations of the recombinant cytokines in pg/mL.

### 2.8. Annexin V Staining

To detect the apoptotic cells, FC was carried out by using BD Annexin V Detection Kit I (BD Biosciences). Briefly, both mock and CHIKV infected cells were detached from the cell culture dishes by trypsin-EDTA treatment. The cells were washed twice in ice cold 1× PBS and then resuspended in 100 μL of 1× Annexin V binding buffer at a density of 1 × 10^6^ cells/mL. Then, 2.5 μL each of APC conjugated Annexin V and 7-AAD cocktail was added per sample, gently mixed by vortexing and incubated at RT for 15 min in the dark. After that, 400 µL of 1× Annexin V binding buffer was added per tube, samples were acquired immediately by the BD FACS Calibur^TM^ and analyzed by the CellQuest Pro software. A total of approximately five thousand cells were acquired per sample.

### 2.9. Western Blot Analysis

Protein expression was assessed by Western blot analysis according to the protocol described earlier [55]. In brief, both the mock and CHIKV infected cells were washed once with ice cold 1× PBS and the whole cell lysate (WCL) was prepared by Radio Immuno Precipitation Assay (RIPA) lysis buffer (150 mM NaCl, 1% NP-40, 0.5% sodium deoxycholate, 0.1% SDS, 50 mM Tris, pH 8.0, supplemented with protease inhibitor cocktail) and centrifuged at 13,000 rpm for 15 min at 4 °C. The protein concentration was quantified by the Bradford reagent (Sigma-Aldrich). Equal amount of protein was loaded in the 10%–12% SDS-PAGE after mixing with 2× sample buffer (130 mM Tris-Cl, pH 8.0, 20% (*v*/*v*) Glycerol, 4.6% (*w*/*v*) SDS, 0.02% Bromophenol blue, 2% DTT) at a ratio of 1:1 and blotted on to a PVDF membrane (Millipore, MA, USA). Then the transferred membranes were blocked with 3% BSA followed by overnight incubation with the different primary antibodies, HSP90 (1:1000), cleaved caspase-3 (1:1000), cleaved caspase-8 (1:1000), caspase-9 (C9) (1:1000), β-actin (1:1000) and GAPDH (1: 1000). Then, the membranes were thoroughly washed with TBST for five times and incubated with the HRP conjugated secondary antibodies for 2 h at RT. After washing with TBST for three times, the membranes were subjected to chemiluminescence detection (Immobilon Western Chemiluminescent HRP substrate, Millipore or SuperSignal West Femto reagent, Thermo Scientific, Waltham, MA, USA) by the Bio-Rad gel doc with the Quantity One software (Bio Rad). For band intensity quantification, Western blot images were subjected to further analysis by the Quantity One 1-D analysis software while normalizing to the corresponding loading control.

### 2.10. RT-PCR

For quantitation of CHIKV RNA inside the host cells, Raw264.7 cells were infected with DRDE-06 strain of CHIKV with and without 17-AAG as mentioned previously. Cells were harvested at 8 hpi and the total RNA was isolated using Trizol reagent (Invitrogen). The RT was performed with equal amount of RNA (1 µg) by using First Strand cDNA synthesis kit (Thermo Scientific, Waltham, MA, USA) as per the manufacturer’s instructions. This cDNA was used to amplify viral non-structural gene (NSP2) using primers (F)-5′CGAGGATCCACTGAATGAAATATGC-3′ and (R)-5′CGACTCGAGTTAACATCCTGCTCGGGTGG-3′; structural gene (E1) using primers (F)-5′TGCCGTCACAGTTAAGGACG3′ and (R)-5′CCTCGCATGACATGTCCG3′ through RT-PCR, along with GAPDH as housekeeping gene in Eppendorf master cycler pro S. The RT-PCR products were subjected to 1.5% agarose gel electrophoresis and GAPDH served as internal amplification control. Relative band intensities were calculated with the help of the Image J software (NIH, Bethesda, MD, USA) for the respective amplification.

### 2.11. Statistical Analysis

Statistical Analysis was performed using the GraphPad Prism 5.0 software (GraphPad Software Inc., San Diego, CA, USA). Data are represented as Mean ± SEM. The comparison between the groups was performed by the 2 way ANOVA with Bonferroni post-hoc test unless otherwise mentioned. Data presented here are representative of at least three independent experiments. *p* < 0.05 is considered as statistically significant difference between the groups.

## 3. Results

### 3.1. Determination of CHIKV Infection in Macrophages

**Figure 1:**
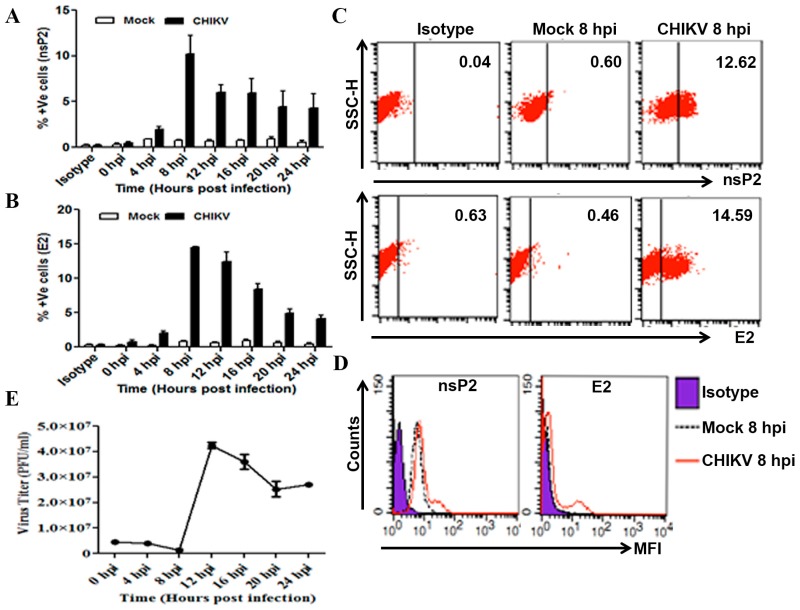
Infection of Chikungunya virus in macrophages. The Raw264.7 cells in 6 well plate were in vitro infected with CHIKV at MOI 5 for different time points or as described in the materials and methods section. The infected cells were harvested and stained with respective antibodies against the viral proteins followed by FC analysis. Representative bar diagram showing the time kinetics of percent positive cells for nsP2 (**A**) and E2 (**B**) in mock and CHIKV infected Raw cells at an interval of 4 hpi. (**C**) Representative dot plot analysis showing the expression of nsP2 (upper) and E2 (lower), along with isotype control (left), mock (middle) and CHIKV infected (right) macrophages at 8 hpi. (**D**) Mean fluorescence intensity (MFI) of nsP2 and E2 at 8 hpi representing the isotype (purple filled), mock (black dashed line) and CHIKV infected (red solid line) macrophage cells. (**E**) Line diagram of viral plaque forming units (PFU/mL), determined by plaque assay from CHIKV infected macrophage cell culture supernatants collected at different time points. Data represent mean ± SEM of three independent experiments. *p* < 0.05 was considered as statistically significant difference between the groups.

In order to establish CHIKV infection, Raw264.7 and CHIKV strain, DRDE-06 were used at MOI 5. The infected cells along with the supernatants were harvested at an interval of 4 h from 0 to 24 hpi. The cells were processed for ICS to detect CHIKV specific antigens, nsP2 and E2 at different time points. Next, the expression pattern of these two CHIKV proteins was assessed by FC and it was observed that, both nsP2 and E2 were detected as early as 4 hpi (nsP2: 2.0 ± 0.24 and E2: 2.05 ± 0.22), while the highest level of proteins were noticed at 8 hpi (nsP2: 10.19 ± 2.04 and E2: 14.47 ± 0.17) followed by the gradual decrease at later time points (Figure 1A,B). Accordingly, Raw cells were harvested only at 8 hpi to assess CHIKV infection for subsequent experiments and representative FC data have been given towards the CHIKV replication and infection at 8 hpi (Figure 1C,D). Our current observation indicates that the virus could infect and replicate actively in Raw264.7 cells, with a peak of nsP2 and E2 levels around 8 hpi.

Next, to determine the release of the new infectious virus particles, plaque assay was performed using the supernatants collected at different hpi from the above experiments. Almost no virus particle was found to be released at 8 hpi, while the viral titer increased significantly to 4.25 × 10^7^ ± 0.12 × 10^7^ PFU/mL at 12 hpi indicating effective replication of CHIKV and release of the newly synthesized virus particles (Figure 1E). Subsequently, the viral count went down slowly at 16, 20 and 24 hpi. Since the supernatant was collected at every 4-h interval, the viral titer reflects the newly generated virus particles that were released in that particular period. Taken together, our data show that CHIKV could successfully infect and actively replicate in mouse macrophage cells in vitro.

Since susceptibility of CHIKV infection varies among cell lines with different MOIs in vitro [61], the efficacy of the DRDE-06 strain of CHIKV to infect Raw264.7 cell was also tested at different MOIs (0.1, 1, 5 and 10) at 8 hpi. It was observed that Raw264.7 cell was not susceptible to CHIKV infection at the MOI 0.1 and 1 in vitro as no significant E2 percent positive cells were detected with respect to the mock. However, at the MOI 5 and 10, E2 positive cells were found to be around 15% and 30% respectively (Figure S1A–C). Thus, MOI 5 was used for the rest of our experiments as an optimal virus load to the cells.

Earlier, it was reported that DRDE-06 strain of CHIKV replicates faster than S 27 strain in the Vero cell line [59]. Thus, here the infectivity of the S 27 strain was tested in the Raw264.7 cell line, to demonstrate that CHIKV infection in Raw cells is not strain specific. It was observed that S 27 also infects and replicates in Raw264.7 cells with less infectivity as compared to DRDE-06 strain (Figure S2C). Immune cells are generally found to be resistant to viral infections as compared to other somatic cells [61]. Thus, here the permissiveness of DRDE-06 was compared in Vero cell line. The expression pattern of CHIKV proteins was assessed by FC analysis. It was observed that both nsP2 and E2 were detected as early as 4 hpi (DRDE-06 nsP2: 31.79 ± 2.30 and E2: 85.62 ± 4.67), while the highest level of proteins were noticed at 8 hpi (DRDE-06 nsP2: 74.83 ± 0.52 and E2: 83.84 ± 5.39) followed by the gradual decrease at the later time points (Figure S2A,B). Taken together, our data show that different strains of CHIKV could successfully infect and actively replicate in mouse macrophage cells in vitro. Moreover, the macrophages (Raw264.7 cells) are found to be less permissible to CHIKV than Vero cells.

### 3.2. CHIKV Infection Induces Apoptosis in Macrophages

**Figure 2:**
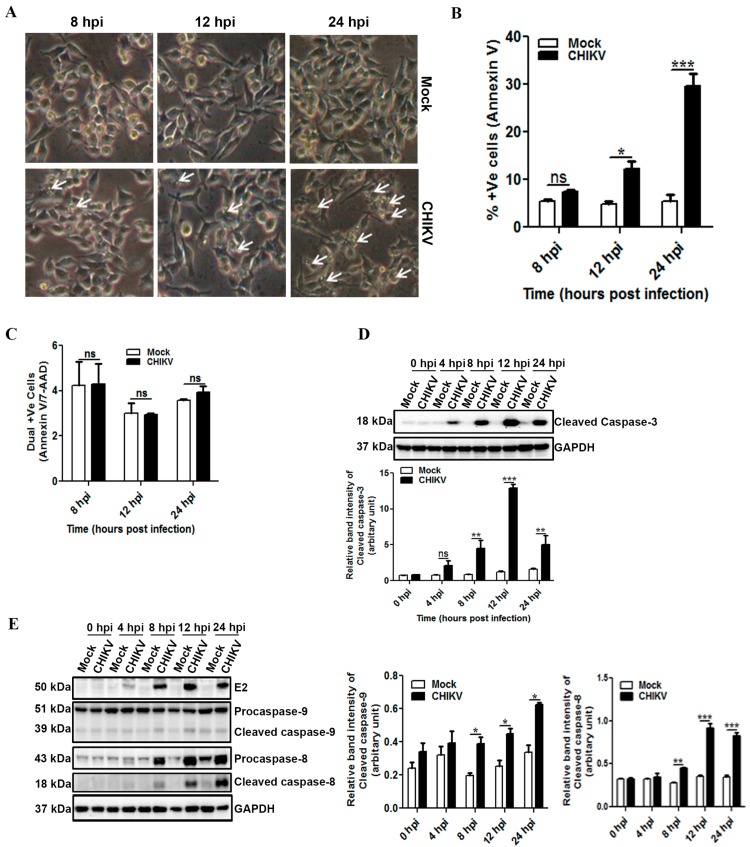
Induction of apoptosis following CHIKV infection in Macrophages. Raw264.7 cells were infected with CHIKV at MOI 5 for different time points as described earlier. Infected cells were viewed under bright field microscope or processed for FC analysis. (**A**) Bright field microscopic images taken at 8, 12 and 24 hpi with 20× magnification. White arrows indicate observed morphological changes in the infected cells. (**B**) Graphical representation showing percent positive cells for Annexin V in mock (white bar) and CHIKV infected samples (dark bar) at 8, 12 and 24 hpi. (**C**) Bar diagram showing percent dual positive cells for Annexin V and 7-AAD in mock (white bar) and CHIKV infected samples (dark bar) at 8, 12 and 24 hpi. (**D**) CHIKV infected Raw cells were harvested, lysed and separated by SDS-PAGE. Western blot was performed and expression pattern of cleaved caspase-3 was assessed at different hpi as shown (upper), bar diagram showing relative band intensity of cleaved caspase-3 (lower). GAPDH was used as loading control. (**E**) Western blot analysis of E2, caspase-9 and caspase-8 (left), bar diagram showing relative band intensity of cleaved caspase-9 and cleaved caspase-8 (right). Data represent mean ± SEM of at least three independent experiments. *p* < 0.05 was considered as statistically significant difference between the groups. (* *p* < 0.05; ** *p* ≤ 0.01; *** *p* ≤ 0.001).

Apoptosis of virus infected cell is known to bear one of the important consequences towards viral replication, dissemination of the virus particle to the neighboring host cells as well as antigen presentation [62,63,64]. CHIKV infection has been recently reported to induce apoptosis in host epithelial cells [55,61,64]. However, apoptosis in host macrophages during CHIKV infection has not been reported yet. To assess whether mouse macrophages undergo apoptosis post CHIKV infection, the Raw cells were inoculated with CHIKV and processed at different time intervals for subsequent analysis. The bright field microscopic images showed the development of CPE at 8 hpi in CHIKV infected Raw cells (Figure 2A). Furthermore, visible tiny cell blebbings were observed at 12 hpi followed by few rounding and detachment of cells at 24 hpi. However, no such morphological changes were observed in the mock. The observed characteristic features of the cells indicate that the cells might have undergone apoptotic process after CHIKV infection. In order to confirm apoptosis in macrophages during CHIKV infection, the mock and the infected cells were stained with Annexin V and 7-AAD at different hpi. The FC analysis depicted a very small fraction of cells to be positive for Annexin V in both the mock (6.2 ± 0.5) and the CHIKV (7.55 ± 0.36, *p* > 0.05) infected cells at 8 hpi (Figure 2B). However, a significant increase in the population of Annexin V positive cells was observed at 12 hpi (Mock; 5.6 ± 0.9 vs. CHIKV; 8.67 ± 0.49, *p* < 0.01) and at 24 hpi (Mock; 5.3 ± 0.06 vs. CHIKV; 17.7 ± 0.05, *p* < 0.001) (Figure 2B). Interestingly, both the Annexin V and the 7-AAD dual positive cells were not increased significantly with time in the CHIKV infected macrophages as compared to the mock (Figure 2C), which confirms that CHIKV induces apoptotic marker, Annexin V without inducing necrosis. To further confirm the induction of apoptosis, the infected cells were harvested at 0, 4, 8, 12 and 24 hpi. Western blot analysis was performed to detect apoptosis through cleaved caspase-3. Surprisingly, the induction of cleaved caspase-3 was observed as early as 4 hpi in the case of CHIKV infected samples. Moreover, the expression of cleaved caspase-3 at 8 and 12 hpi in the CHIKV infected cells was significantly higher than the corresponding mock (Figure 2D). Hence, our data suggest that CHIKV infection induces apoptosis in macrophages in a time dependent manner.

To investigate the in-depth pathway of cleaved caspase-3 induction by CHIKV in macrophages, expression of both cleaved caspase-9 and cleaved caspase-8 was assessed by Western blot analysis. It was observed that both cleaved caspase-9 and -8 were induced during CHIKV infection in the macrophages (Figure 2E). This suggests that CHIKV might induce apoptosis in macrophages through intrinsic as well as extrinsic pathways. Hence, investigation was carried out to assess the importance of apoptosis in CHIKV infected macrophages using a pan caspase inhibitor, Z-VAD-FMK. First, the cytotoxicity of Z-VAD-FMK was assessed by MTT assay with different concentrations. As shown in Supplementary Figure S3A, around 100% and 90% cells were viable after 24 h with 40 µM and 80 µM concentrations of Z-VAD-FMK respectively. Thus, 40 µM concentration of Z-VAD-FMK was used for further studies. The CHIKV infection was assessed in Raw cells in the presence of Z-VAD-FMK by FC analysis. It was observed that, Z-VAD-FMK did not suppress the CHIKV E2 percent positive cells significantly (Figure S3B). Surprisingly, Z-VAD-FMK did reduce the newly synthesized CHIKV progenies around 2.5 fold (Figure S3C). This suggests that Z-VAD-FMK might not interfere in the CHIKV protein expression but it can reduce CHIKV infection by reducing the release of new virus particles in the host macrophages. Since CHIKV induced phosphatidyl serine in the host macrophages (Figure 2B), the effect of Z-VAD-FMK on Annexin V binding was also assessed. It was found that, Z-VAD-FMK reduced the Annexin V binding by 35% (Figure S3D) with 40 µM concentration. Moreover, the treatment of Z-VAD-FMK was found to reduce the inductions of cleaved caspase-3, cleaved caspase-9 and cleaved caspase-8 (Anisomycin treatment was used as positive control for cleaved caspase-9 and -8, [65]) during CHIKV infection in the host macrophages (Figure S3E,F). Taken together, the result suggests that inhibition of apoptosis by Z-VAD-FMK significantly affects CHIKV infection in macrophages, without altering virus protein expression.

### 3.3. CHIKV Infection Upregulates Pro-Inflammatory Response in Mouse Macrophages

**Figure 3:**
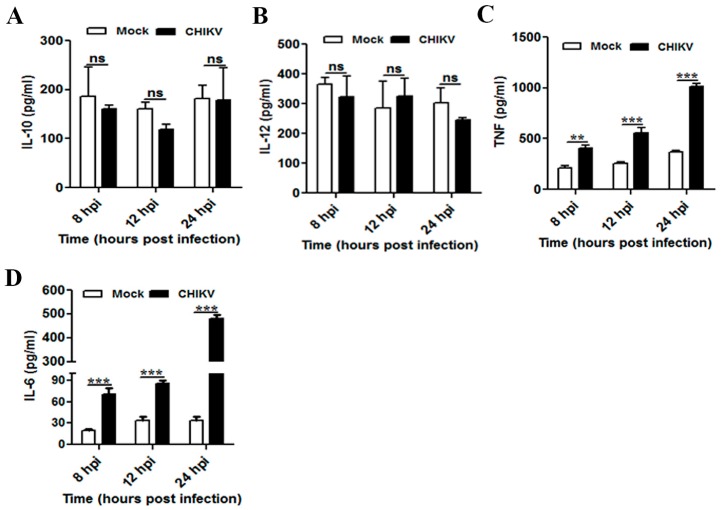
Modulation of macrophage derived cytokines during CHIKV infection. Raw264.7 cells were infected with CHIKV at MOI 5. The cell culture supernatants were collected at different time points and the levels of secreted cytokines in the samples were quantified. Graphical representation showing the amount of secreted: IL-10 (**A**); IL-12 (**B**); TNF (**C**); and IL-6 (**D**) in mock and CHIKV infected macrophage as quantified using sandwich ELISA. Data represent mean ± SEM of at least three independent experiments. *p* < 0.05 was considered as statistically significant difference between the groups. (ns, non-significant; ** *p* ≤ 0.01; *** *p* ≤ 0.001).

To determine the physiological and functional relevance of macrophages during CHIKV infection, cell culture supernatants were collected at different time points (8, 12, and 24 hpi) and quantified for the secreted cytokines (e.g., IL-10, IL-12, TNF and IL-6) and chemokine, MCP-1 by ELISA. No significant changes were observed in both IL-10 and IL-12 cytokines in CHIKV infected cells as compared to mock in all the time points (Figure 3A,B). It was observed that TNF was upregulated at 8 hpi 405 ± 33 pg/mL (mock 211 ± 20 pg/mL, *p* ≤ 0.01) and followed by a maximum peak at 24 hpi 1016 ± 30 pg/mL (mock 367 ± 15 pg/mL, *p* ≤ 0.001) (Figure 3C). The production of IL-6 was found to be upregulated as early as 8 hpi (mock 20 ± 2 pg/mL, CHIKV 71 ± 8 pg/mL, *p* ≤ 0.001) and followed by a maximum peak at 24 hpi (mock 34 ± 6 pg/mL, CHIKV 480 ± 17 pg/mL, *p* ≤ 0.001) (Figure 3D). Together, it was found that both TNF and IL-6 were increased during CHIKV infection in Raw cells in a time dependent manner.

The role of MCP-1 has been shown as an important mediator of inflammation in a variety of diseases, which recruit other immune cells to the site of impact to induce inflammatory responses [66,67]. Monocytes and macrophages are one of the major sources of MCP-1. Accordingly, it has been assessed whether CHIKV infection induces MCP-1 production in macrophages. It was observed that there was no significant difference in MCP-1 production during CHIKV infection at early time points (8 and 12 hpi) as compared to mock. However, CHIKV infection positively modulated MCP-1 secretion around 24 hpi (1354 ± 19 pg/mL) as compared to mock (1114 ± 64 pg/mL, *p* < 0.05). This result indicates that CHIKV infection in macrophages may upregulate TNF, IL-6 and MCP-1 over time, while no such significant changes were observed for IL-10 and IL-12.

### 3.4. Induction of MHCs and Co-Stimulatory Molecules in CHIKV Infected Macrophages

**Figure 4:**
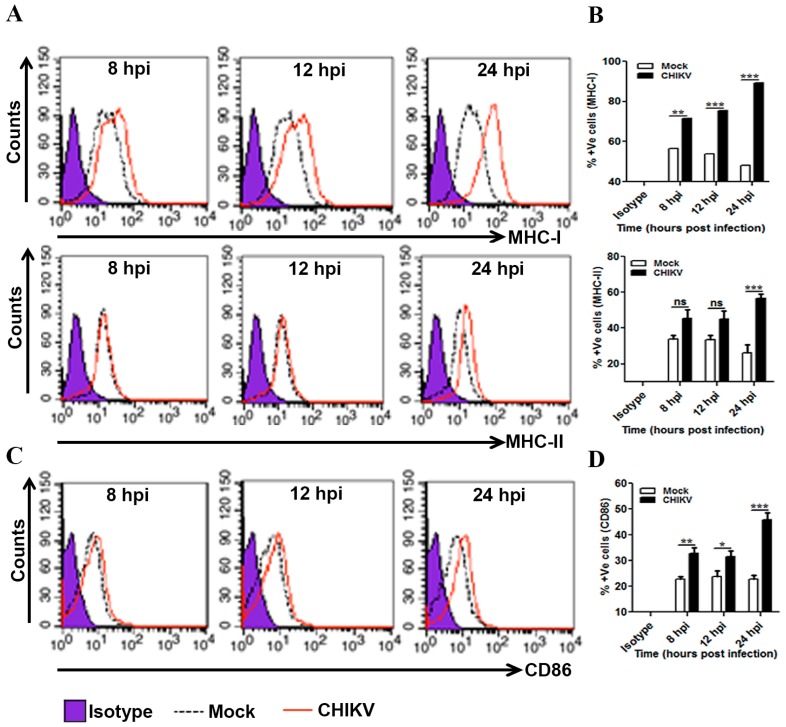
Expression pattern of MHC and co-stimulatory molecules during CHIKV infection in macrophages. CHIKV infected Raw264.7 cells were harvested at different time intervals followed by flow cytometry based analysis. (**A**) MFI representing MHC-I (upper panel) and MHC-II (lower panel) expressions in isotype (purple filled), mock (black dashed line) and CHIKV infected macrophage (red solid line). (**B**) Bar diagram showing percent positive cells for MHC-I (upper panel) and MHC-II (lower panel) in mock (white bar) and CHIKV infected samples (dark bar) at 8, 12 and 24 hpi. (**C**) MFI representing CD86 expression in isotype (purple filled), mock (black dashed line) and CHIKV infected macrophage (red solid line). (**D**) Bar diagram showing percent positive cells for CD86 in mock (white bar) and CHIKV infected samples (dark bar) at 8, 12 and 24 hpi. Data represent Mean ± SEM of at least three independent experiments. *p* < 0.05 was considered as statistically significant difference between the groups. (ns, non-significant; * *p* ≤ 0.05; ** *p* ≤ 0.01; *** *p* ≤ 0.001).

APCs like macrophages are known to show altered expression of MHCs and co-stimulatory molecules (e.g., B7 molecules) during pathogenic encounter including viral infection [68,69,70,71]. Flow cytometry based investigations were carried out to detect the surface expression of MHC-I, MHC-II and inducible co-stimulatory molecule, CD86 (B7.2) in the CHIKV infected macrophages at different time points. It was found that the MHC-I surface expression was significantly up-regulated at 8, 12 and 24 hpi (Figure 4A,B). Unlike MHC-I, the surface expression of the MHC-II was increased significantly only at the later time point (24 hpi) after CHIKV infection (Figure 4A,B). Similar to MHC-I, the expression level of CD86 was found to be upregulated at different hpi (Figure 4C,D). This result indicates that CHIKV infection may significantly induce MHCs and co-stimulatory molecules in mouse macrophages.

### 3.5. Regulation of CHIKV Infection by 17-AAG in Macrophages

**Figure 5:**
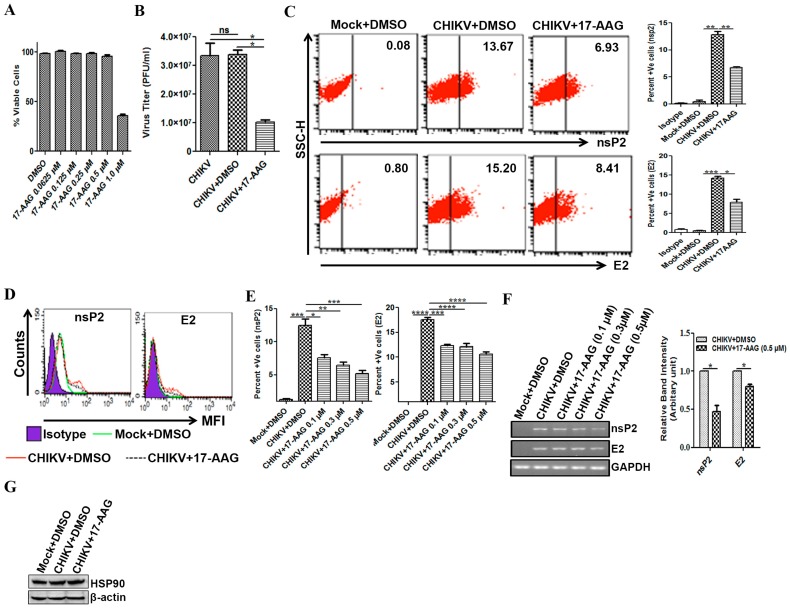
Modulation of CHIKV infection by 17-AAG in macrophages. Raw cells were infected with CHIKV at MOI 5. The cells were treated with either DMSO or 17-AAG as described earlier. (**A**) Percent viable cells treated with different concentrations of 17-AAG with respect to the solvent control (DMSO) as determined by MTT assay. (**B**) Line diagram showing CHIKV titer as PFU/mL in CHIKV, CHIKV + DMSO and CHIKV + 17-AAG (0.5 µM) at 12 hpi. (**C**) Representative dot plot analysis depicting percent positive cells for nsP2 and E2 at 8 hpi. Bar diagram showing percent positive cells for nsP2 (upper panel) and E2 (lower panel) in different samples at 8 hpi. (**D**) MFI representing expression of nsP2 and E2 in isotype (purple filled), mock + DMSO (green solid line), CHIKV + DMSO (red solid line) and CHIKV + 17AAG (black dashed line) at 8 hpi. (**E**) Graphical representation showing percent positive cells for nsP2 (left) and E2 (right) at 0.1 µM, 0.3 µM and 0.5 µM 17-AAG treatments at 8 hpi. (**F**) Agarose gel picture showing RT-PCR of nsP2 and E2 (left) and bar diagram (right) showing reduction in the CHIKV RNA synthesis in Raw cells after 0.5 µM 17-AAG treatment at 8 hpi. (**G**) Representative Western blot showing HSP90 level for mock + DMSO, CHIKV + DMSO and CHIKV + 17-AAG at 8 hpi. Data represent mean ± SEM of at least three independent experiments. *p* < 0.05 was considered as statistically significant difference between the groups. (ns, non-significant; * *p* < 0.05; ** *p* ≤ 0.01; *** *p* ≤ 0.001).

Our recent study showed that CHIKV nsP2 is stabilized by HSP90 during the active stage of replication in the Vero cells, which was abrogated by HSP90 inhibitor, GA [55]. Here, 17-AAG, a less toxic derivative of GA [72], was employed to study its effect on CHIKV infection in macrophages. The cytotoxicity of different concentrations of 17-AAG (0.0265, 0.125, 0.25, 0.5 and 1.0 µM) was tested on Raw cells by MTT assay for 24 h. It was observed that nearly 98% cells were viable up to 0.5 µM concentrations of 17-AAG (Figure 5A). However, with 1 µM of 17-AAG, approximately 50% cells were viable. Hence, 0.5 µM concentration of 17-AAG was selected as a non-cytotoxic dose for further study to work with the viable cells.

The Raw264.7 cells were infected with CHIKV in the presence of either DMSO or 0.5 µM concentration of 17-AAG and the cells were maintained along with the drug until harvesting. Then, the cells and supernatants were collected at 8, 12, 24 hpi and plaque assay was performed. It was observed that the treatment of 17-AAG reduced the number of new viral progeny production by 3.3 fold (*p* < 0.05) as compared to DMSO control (Figure 5B). Subsequently, the cells were collected to estimate the nsP2 and E2 protein levels. FC analysis showed that 17-AAG treatment (0.5 µM) was found to inhibit the level of both the viral proteins by 50% (Figure 5C,D). Furthermore, the dose kinetics depicts that 17-AAG inhibits nsP2 expression around 39% at 0.1 µM concentration, whereas it was further reduced to 58% with 0.5 µM concentration (Figure 5E). In case of the E2 protein, the expression was reduced to 30% at 0.1 µM concentration, however, it was further reduced to 40% with 0.5 µM of 17-AAG. This observation confirms that 17-AAG inhibits CHIKV protein synthesis and new viral progeny production in macrophages. Moreover, in this study it was explored whether 17-AAG treatment can reduce the level of CHIKV RNA. It was observed that the RNA levels were reduced by 2 fold (*p* < 0.05) for nsP2 and 1.25 fold (*p* < 0.05) for E2 only in the presence of 0.5 µM of 17-AAG (Figure 5F). This observation suggests that 17-AAG may reduce the production of viral progeny by inhibiting both the levels of nsP2 and E2 proteins as well as RNA. The result also indicates that all the concentrations of 17-AAG were able to suppress the viral protein levels remarkably, whereas the RNA levels were reduced significantly only with 0.5 µM concentration.

Next, the effect of 17-AAG treatment towards HSP90 expression in mock and CHIKV infected Raw cells were investigated. The Western blot analysis showed that expression of HSP90 remained unchanged in CHIKV + DMSO and CHIKV + 17-AAG treated macrophages as compared to Mock + DMSO. This suggests that 17-AAG may regulate CHIKV infection by abrogating HSP90 activity without modulating its expression (Figure 5G).

### 3.6. 17-AAG Regulates Apoptosis and Cellular Immune Responses during CHIKV Infection in Macrophages

**Figure 6:**
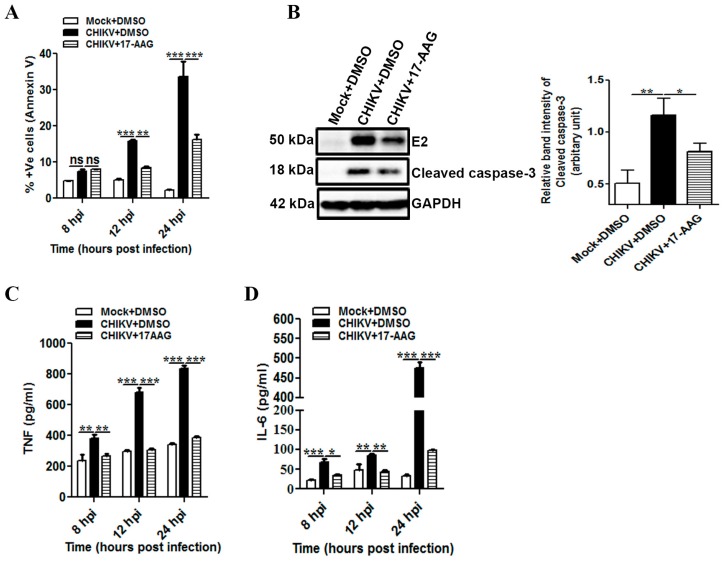
Suppression of CHIKV induced apoptosis and pro-inflammatory responses in macrophages by 17-AAG. Raw cells were infected with CHIKV at MOI 5. The cells were treated with either DMSO or 17-AAG as described earlier. (**A**) Percent Annexin V positive cells for mock + DMSO, CHIKV + DMSO and CHIKV + 17-AAG at 8, 12 and 24 hpi. (**B**) Western blot analysis showing the expression of the cleaved caspase-3, E2 and GAPDH in the presence of 17-AAG during CHIKV infection in macrophage at 12 hpi (left). Bar diagram showing relative band intensity of cleaved caspase-3 of mock + DMSO, CHIKV + DMSO and CHIKV + 17-AAG analyzed by the Quantity One 1-D analysis software (right). Cell culture supernatants were collected and were assessed for TNF (**C**) and IL-6 (**D**) secretion for mock + DMSO, CHIKV + DMSO and CHIKV + 17-AAG by sandwich ELISA at 8, 12 and 24 hpi. Data represent mean ± SEM of at least three independent experiments. *p* < 0.05 was considered as statistically significant difference between the groups. (ns, non-significant; * *p* < 0.05; ** *p* ≤ 0.01; *** *p* ≤ 0.001).

Since apoptosis was detected by Annexin V binding during CHIKV infection (Figure 2A), experiments were performed to test whether 17-AAG can regulate CHIKV induced apoptosis and cellular immune responses in macrophages. It was observed that the Annexin V positive cells were 33.68% ± 4.15% after CHIKV infection, which was reduced to 18.43% ± 1.52% with 17-AAG at 24 hpi (Figure 6A). Furthermore, quantitative Western blot analysis showed that CHIKV induced cleaved caspase-3 upregulation was found to be reduced by around 30% with 17-AAG treatment as compared to the DMSO control at 12 hpi (Figure 6B, left and right panel). Together, it appears that 17-AAG treatment might regulate CHIKV induced apoptosis of host macrophages.

Since pro-inflammatory responses such as IL-6, TNF and MCP-1 were significantly induced in CHIKV infected macrophages (Figure 3), experiments were carried out to assess the efficacy of 17-AAG to suppress the cytokine and chemokine induction. The data showed that TNF level was 683 ± 26 pg/mL and 838 ± 18 pg/mL in CHIKV + DMSO at 12 and 24 hpi respectively, whereas, the levels were reduced to 304 ± 12 pg/mL and 385 ± 10 pg/mL in the presence of 17-AAG (Figure 6C). Similarly, the IL-6 levels were 85 ± 6 pg/mL and 476 ± 15 pg/mL in CHIKV + DMSO at 12 and 24 hpi respectively, whereas, the levels were reduced to 44 ± 3 pg/mL and 98 ± 2 pg/mL in the presence of 17-AAG (Figure 6D). The present data showed that TNF was reduced significantly (around 50%) upon 17-AAG treatment as compared to DMSO control. Similarly, the reduction of IL-6 after 17-AAG treatment was found to be around 50% at 8 and 12 hpi, whereas at 24 hpi it reached up to 80% as compared to DMSO control. In addition, the modulation of MCP-1 during CHIKV infection was also reduced after 17-AAG treatment (CHIKV+DMSO 1399 ± 15 pg/mL, CHIKV+17-AAG 641 ± 7 pg/mL, *p* ≤ 0.001).

Unlike pro-inflammatory cytokine and chemokine production, inductions of MHC-I, MHC-II and CD86 by CHIKV were not suppressed upon 17-AAG treatment in all the time points (Figure S4). Together, the data indicate that 17-AAG might down regulate pro-inflammatory cytokine/chemokine production of host macrophages, without altering the induced immune activation markers like MHCs and CD86 during infection.

## 4. Discussion

CHIKV has been known to infect a wide range of cells including monocytes/macrophages, [22,36,61,73] and elicits strong immune response which involves the production of anti-viral IFNs, pro-inflammatory cytokines, chemokines and other growth factors [22,23,24,25,26,28,29,30]. The current study provides evidence that there is an alteration in immune responses of mouse macrophages (Raw264.7 cell line) comprising MHCs, co-stimulatory molecule (CD86), major pro-inflammatory cytokines/chemokine and host cell apoptosis during CHIKV infection in vitro. Furthermore, we report that 17-AAG, a potential HSP90 inhibitor, may effectively regulate CHIKV infection, apoptosis and associated inflammatory responses of host macrophages.

Acute phase CHIKV infection in human blood monocytes was found to induce robust and rapid innate immune responses [36]. In the current study, the CHIKV infected macrophages showed an increased level of viral proteins (both nsP2 and E2) at 8 hpi and maximum release of new viral progenies at 12 hpi. Like other Alphaviruses, it was reported that CHIKV infection induces apoptosis and CPE in other cell types [23,55,74,75,76,77]. However, induction of apoptosis in CHIKV infected macrophages is yet to be defined. Here, CHIKV infected macrophages showed characteristic features of an apoptotic cell in addition to CPE, membrane blebbings, rounding off and detachment. The results showed increased surface binding of Annexin V and increased cleaved caspase-3, affirming the induction of apoptosis in the CHIKV infected macrophages with MOI 5. Furthermore, it was demonstrated for the first time that CHIKV induces apoptosis in macrophages by both intrinsic and extrinsic pathways, as induced expression of the cleaved caspase-9 and -8 were observed during infection. Earlier, apoptosis was not detected in mouse macrophages infected with CHIKV at MOI 1 [22]. The differences in the observations could be due to the differences in MOIs being used in their experiments, different strain of viruses as well as different experimental set up. Apoptosis is an important anti-viral mechanism and is also considered to have a protective role in macrophages to ward off the viruses, thereby impairing viral propagation [78,79]. Interestingly, the treatment of Z-VAD-FMK was found to reduce new viral progeny release of CHIKV and infection, without altering the frequency of E2 percent positive host macrophages. The importance of Z-VAD-FMK on CHIKV infection was also studied earlier in HeLa cells, suggesting the reduction of both CHIKV replication and infection in vitro [64]. Moreover, Z-VAD-FMK was also found to differentially regulate other viral replication and infection [80,81,82,83]. Arguably, our study in macrophages may highlight the importance of CHIKV-induced apoptosis in increasing the viremia and propagation of infection to the nearby host cells. Further investigations are required to understand the mechanism in details.

CHIKV infection associated inflammatory response involves production and secretion of several pro-inflammatory cytokines. However, predominance of anti-inflammatory responses might also prevail under such conditions, which are against a common description and the notion of CHIKV infection [30]. IL-12 and TNF possess potent anti-viral property and also promote macrophage activation [84,85,86], while IL-10 plays an important cross-regulatory role during infection associated inflammation [87]. Moreover, IL-6 and MCP-1 induction during CHIKV infection have been shown to promote inflammatory and heightened cellular immune responses [30,66]. In this study, we assessed the expression of macrophage derived pro-inflammatory cytokines and found that there was a time-dependent increase in the secreted TNF, IL-6 and MCP-1 during CHIKV infection, while the production of IL-10 and IL-12 remain unchanged.

Viral infection in APCs like macrophages is known to enhance antigen processing and presentation via MHC to elicit adaptive immune responses. However, some viruses are known to manipulate the expression of MHC and co-stimulatory molecules to evade host cell immunity [32,68,69,70,71]. Moreover, our recent in silico analysis has identified several highly conserved CHIKV specific immunodominant MHC-I restricted peptide epitopes which may elicit strong anti-CHIKV CD8^+^ T cell responses [88]. The present study demonstrated that the levels of MHC-I, MHC-II and CD86 activation markers were elevated in the CHIKV infected macrophages at various time intervals. It is plausible that the CHIKV induced MHCs may present specific immunodominant peptides and also induce CD86 expression in macrophages towards concomitant anti-CHIKV specific T cell immunity, which needs further investigations.

Host derived endogenous HSP90 has been identified to play a critical role in the pathogenesis of CHIKV [55,56]. In this study, we used 17-AAG on CHIKV-infected macrophages and our results demonstrated that the treatment of 17-AAG has reduced the production of live infectious virus particles significantly. Concurrently, there was reduction in the levels of viral proteins (nsP2 and E2) and synthesis of infectious virus particles in the 17-AAG treated-cells indicating its regulatory role during CHIKV infection in macrophages.

Subsequently, the efficacy of 17-AAG was tested towards the regulation of apoptosis in CHIKV-infected macrophages, if any. Although 17-AAG is known to induce cleaved caspase-3 in some cancerous cells [89,90], interestingly, in the current investigation, it was noticed that non-cytotoxic dose of 17-AAG abrogated CHIKV induced apoptosis (measured by Annexin V binding and the expression of cleaved caspase-3). This observation might be explained by the fact that the reduction of CHIKV induced Annexin V and cleaved caspase-3 by 17-AAG might be due to reduction of CHIKV infection in the host macrophages. Our current observation also suggests that the upregulated level of pro-inflammatory cytokines (TNF and IL-6) and chemokine (MCP-1) in CHIKV infected macrophages could be down regulated by 17-AAG treatment, while preserving the expression of MHC and co-stimulatory molecules in macrophages, probably to strengthen the subsequent anti-viral immune responses.

**Figure 7:**
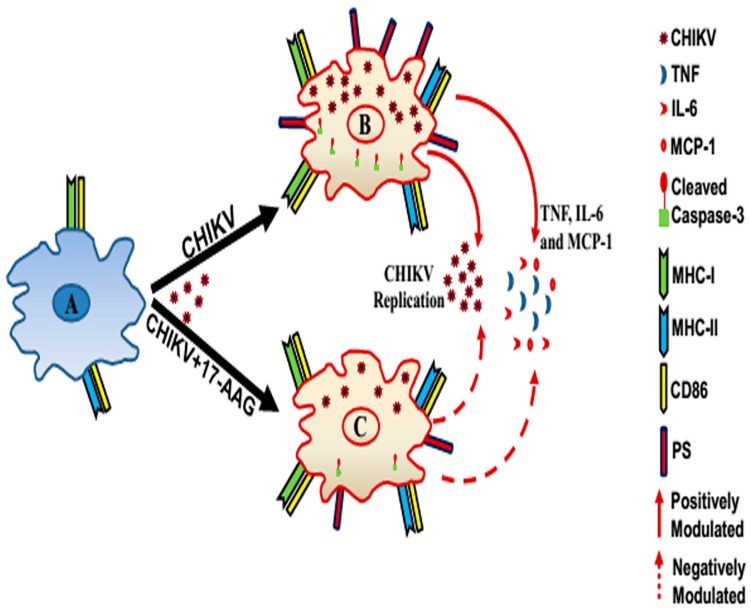
Proposed working model depicting CHIKV infection in macrophages and 17-AAG mediated possible regulation of its altered immune responses by inhibition of HSP90. Uninfected macrophage (**A**); and CHIKV infected macrophage (**B**) showing induction of MHC I/II, CD86 molecules as immune activation markers along with inflammatory cytokines/chemokine (TNF, IL-6 and MCP-1) production and apoptosis by phosphatidylserine (PS) and cleaved caspase-3 expression. (**C**) 17-AAG has been found to regulate the viral infection, apoptosis and inflammatory responses (TNF, IL-6 and MCP-1), suggesting its therapeutic implication in CHIKV infection.

In conclusion, for the first time, we showed that CHIKV infection induces apoptosis, enhances MHCs and co-stimulatory molecule expressions along with IL-6 and MCP-1 production in mouse macrophages, in vitro. We have also identified an important role of 17-AAG to regulate CHIKV infection, pro-inflammatory cytokine/chemokine production and apoptosis of macrophages during viral infection as schematically summarized in Figure 7. Further studies are required to substantiate the mechanism of CHIKV infection in macrophage associated cellular pathways towards apoptosis, induction of host cell immunity and pathogenesis for designing rationale therapeutic drugs against CHIKV infection.

## Supplementary Materials

The following are available online at www.mdpi.com/1999-4915/9/1/3/s1, Figure S1: Characterization of DRDE-06 strain of CHIKV in Raw264.7 cells at different MOIs, Figure S2: Infection pattern of S 27 and DRDE-06 strains of CHIKV in Vero cell line, Figure S3: Effect of Z-VAD-FMK on CHIKV infection and apoptosis in macrophages, Figure S4: Expression level of MHC and CD86 in CHIKV infected macrophages after 17-AAG treatment.
