# Supplementary material for: Regulation of Viral Replication, Apoptosis and Pro-Inflammatory Responses by 17-AAG during Chikungunya Virus Infection in Macrophages
Source: Viruses. 2017 Jan 6;9(1):3. doi: 10.3390/v9010003 (PMC5294972; doi:10.3390/v9010003)
Supplement: Supplementary file 1 [file viruses-09-00003-s001.docx]

Supplementary Materials: Regulation of Viral Replication, Apoptosis and Pro-Inflammatory Responses by 17-AAG during Chikungunya Virus Infection in Macrophages

Tapas K. Nayak, Prabhudutta Mamidi, Abhishek Kumar, Laishram Pradeep K. Singh,
Subhransu S. Sahoo, Soma Chattopadhyay and Subhasis Chattopadhyay


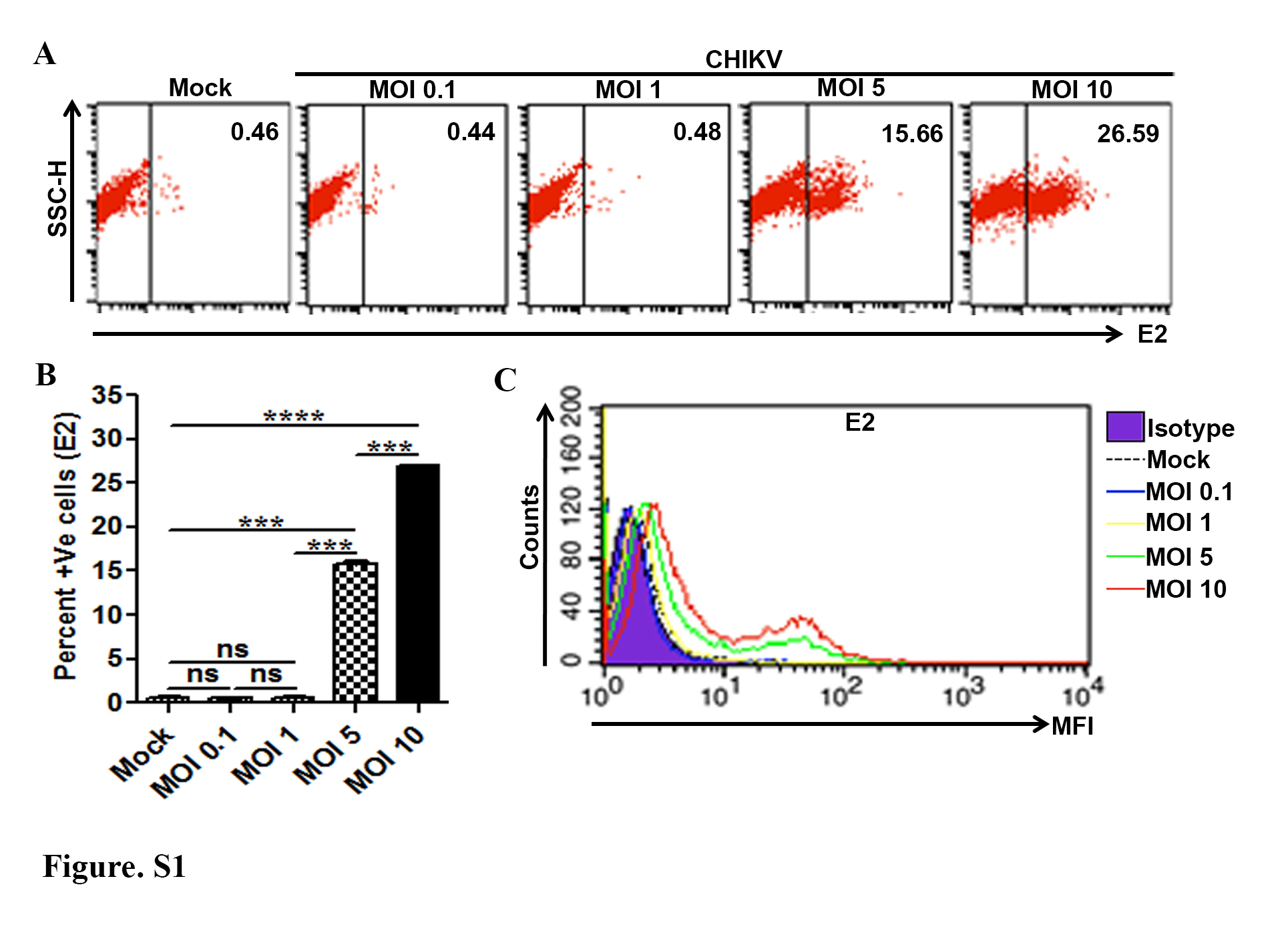


**Figure S1.** Characterization of DRDE-06 strain of CHIKV in Raw264.7 cells at different MOIs. Raw264.7 cells were infected with DRDE-06 strain of CHIKV with different MOIs (0.1, 1, 5 and 10) and expression of E2 was assessed by FC analysis at 8 hpi. (**A**) Representative dot plot analysis showing the expression of E2 protein with mock and CHIKV infected macrophages with 0.1, 1, 5 and 10 MOIs at 8 hpi. (**B**) Graphical representation showing percent positive cells of E2 with 0.1, 1, 5 and 10 MOIs at 8 hpi. (**C**) MFI representing expression of E2 in isotype (purple filled), mock (black dashed line) and CHIKV infected macrophages with different MOIs; 0.1 (blue solid line), 1 (yellow solid line), 5 (green solid line) and 10 (red solid line). Data represent mean ± SEM of at least three independent experiments. *p* < 0.05 was considered as statistically significant difference between the groups. (ns, non-significant; *** *p* ≤ 0.001; **** *p* ≤ 0.0001).


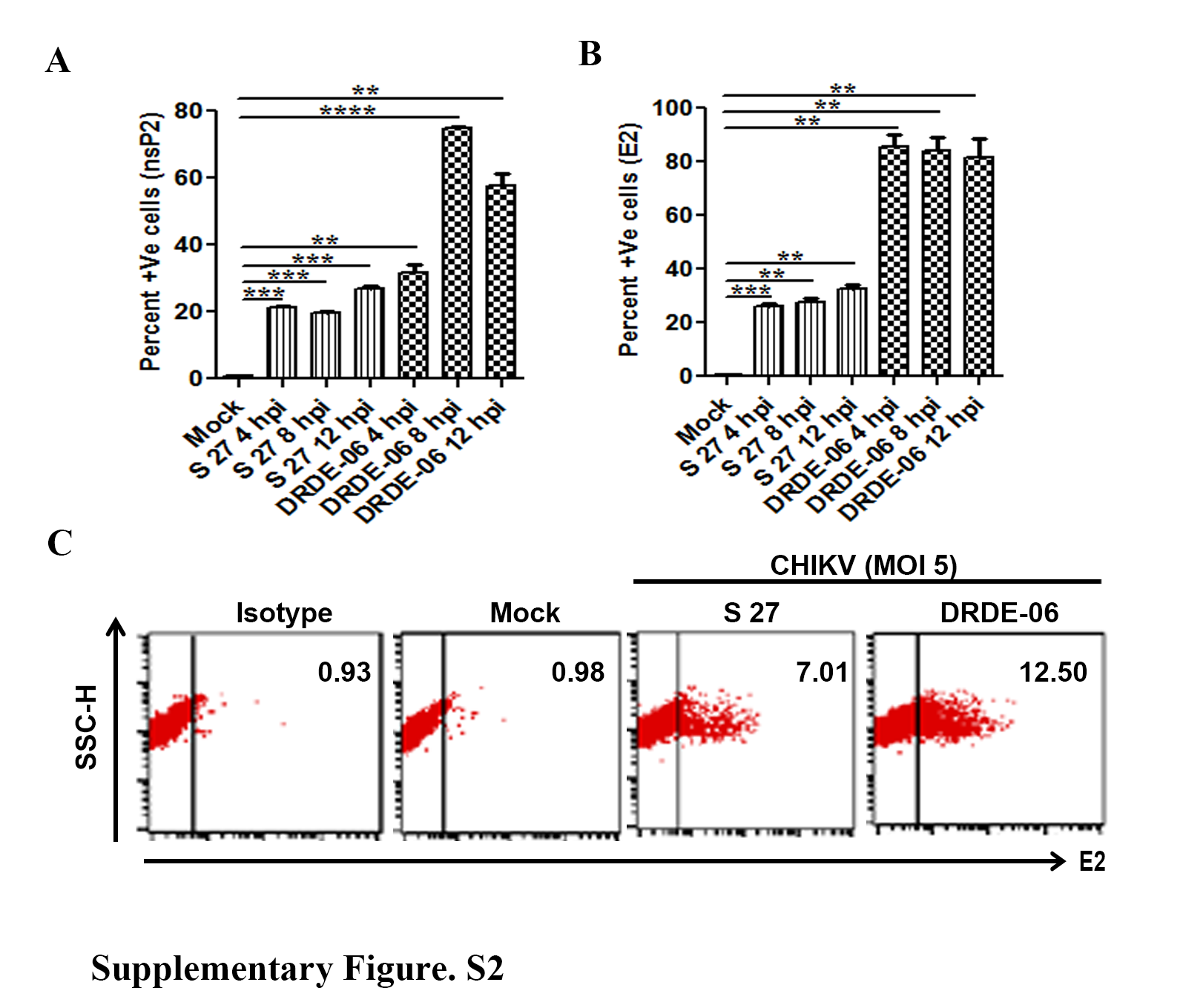


**Figure S2.** Infection pattern of S 27 and DRDE-06 strains of CHIKV in Vero and Raw cell lines. Vero cells were infected with both S 27 and DRDE-06 strains of CHIKV with MOI 5 and expression pattern of nsP2 (**A**) and E2 (**B**) were assessed by FC at 4, 8 and 12 hpi. (**C**) Raw cells were infected with both S 27 and DRDE-06 strains of CHIKV with MOI 5 and expression pattern of E2 was observed by FC analysis at 8 hpi. Data represent mean ± SEM of at least three independent experiments. *p* < 0.05
was considered as statistically significant difference between the groups. (** *p* ≤ 0.01; *** *p* ≤ 0.001;
**** *p* ≤ 0.0001).


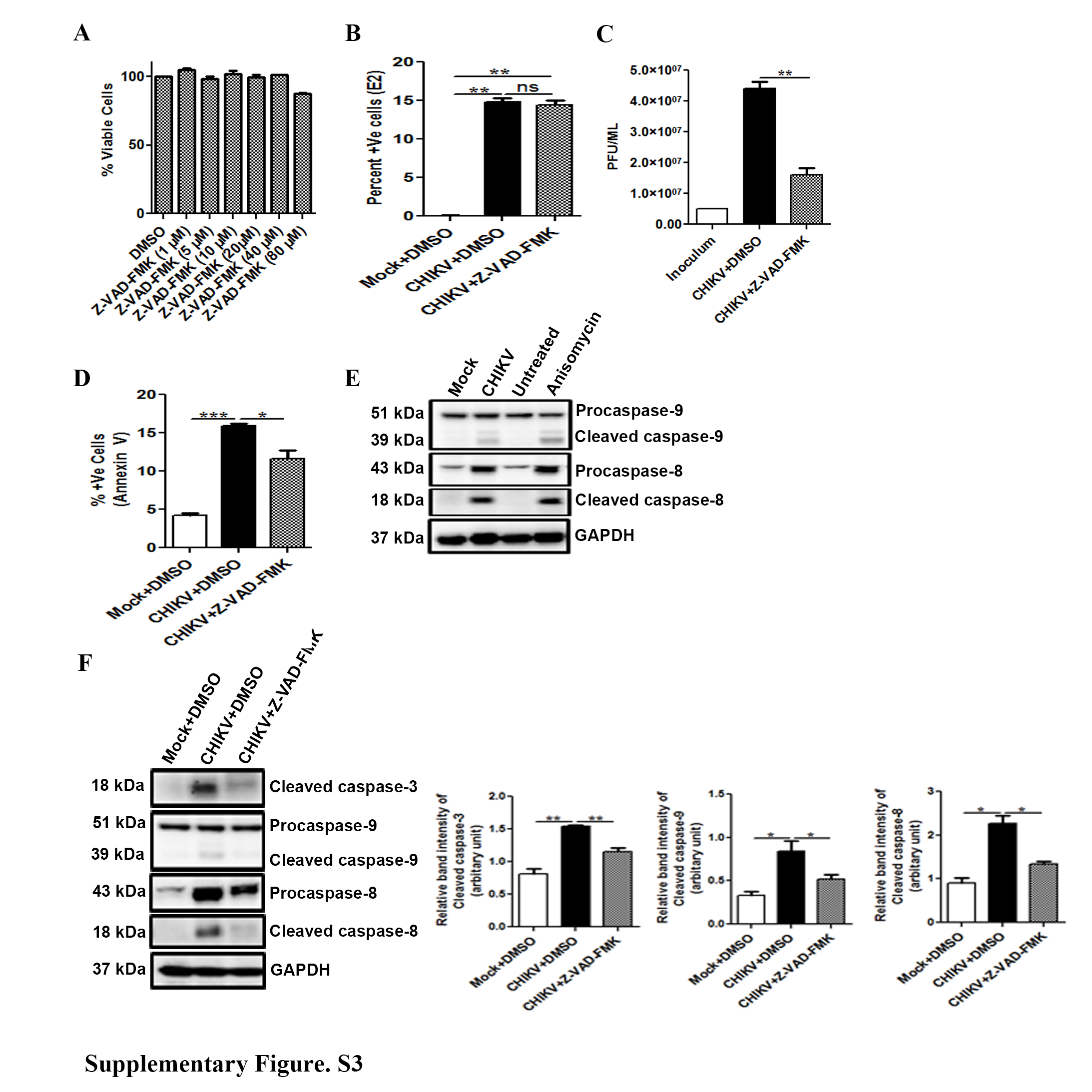


**Figure S3.** Effect of Z-VAD-FMK on CHIKV infection and apoptosis in macrophages: (**A**) Bar diagram showing percent viable cells in the presence of different concentrations of Z-VAD-FMK by MTT assay; (**B**) Cells were infected with DRDE-06 strain of CHIKV with MOI 5 and expression of E2 was assessed by FC analysis at 8 hpi; (**C**) Graphical representation showing virus titer in inoculum, CHIKV + DMSO and CHIKV + Z-VAD-FMK at 12 hpi in macrophages. (**D**) Bar diagram showing percent Annexin V positive cells in Mock + DMSO, CHIKV + DMSO and CHIKV + Z-VAD-FMK at 12 hpi
(**E**) Raw cells were treated with 25 ng/mL concentration of Anisomycin for 3 h to use as a positive control for cleaved caspase-8 and -9 and Western blot was performed with the whole cell lysate along with the CHIKV infected cells (**F**) Representative Western blot analysis showing expression of cleaved caspase-3, -9 and -8 in Mock + DMSO, CHIKV + DMSO and CHIKV + Z-VAD-FMK at 12 hpi (left), representative bar diagram depicting relative band intensities of cleaved caspase-3, -9 and
-8 (right). Data represent mean ± SEM of three independent experiments. *p* < 0.05 was considered as statistically significant difference between the groups. (ns, non-significant; * *p* < 0.05; ** *p* ≤ 0.01;
*** *p* ≤ 0.001).


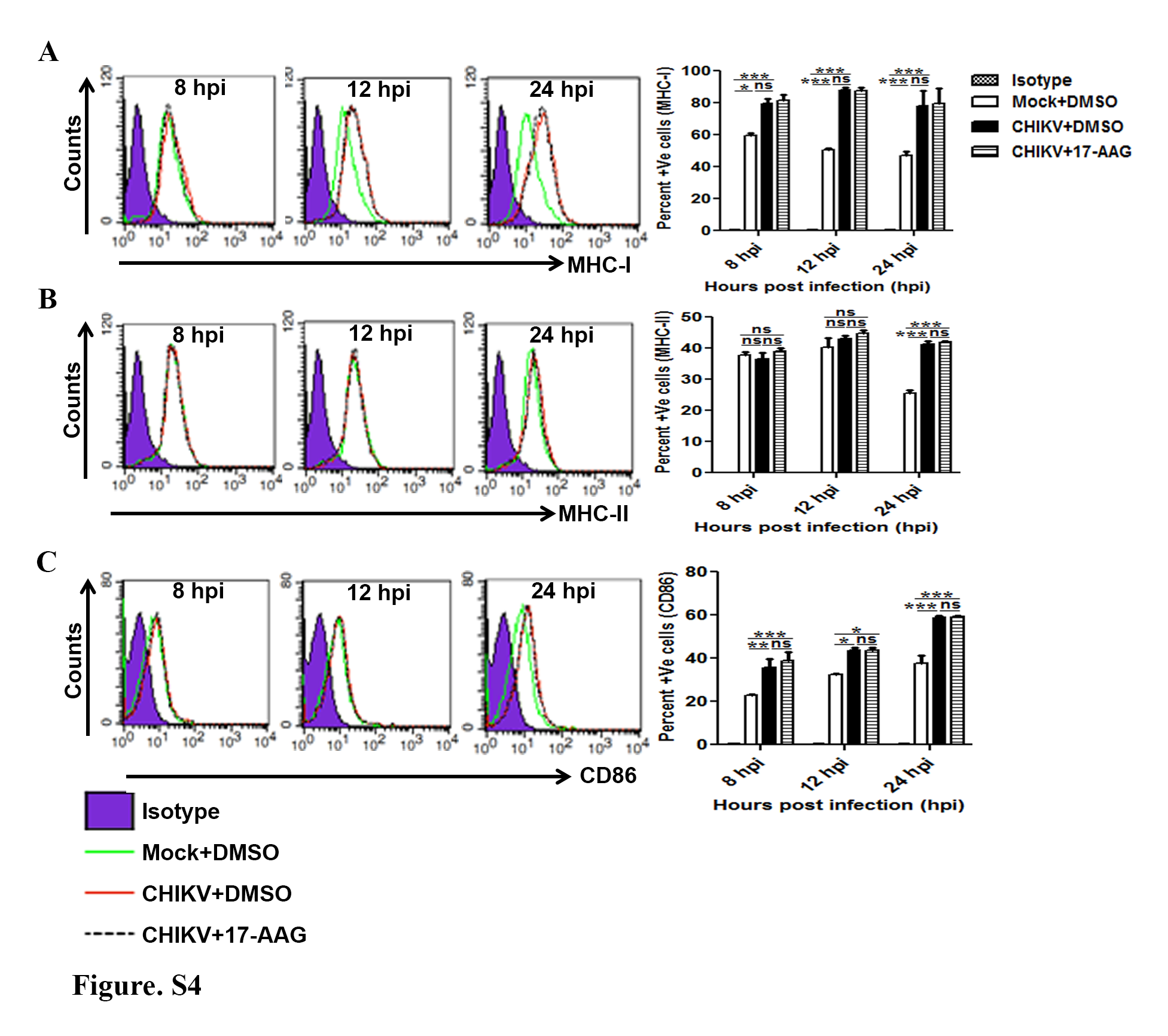


**Figure S4.** Expression level of MHC and CD86 in CHIKV infected macrophages after 17-AAG treatment. CHIKV infected Raw cells were harvested at different time intervals followed by FC based analysis: (**A**) MFI (left) and percent positive cells (right) of MHC-I expression; (**B**) MFI (left) and percent positive cells (right) of MHC-II expression; and (**C**) MFI (left) and percent positive cells (right) of CD86 expression at 8, 12 and 24 hpi. Data represent mean ± SEM of three independent experiments. *p* < 0.05 was considered as statistically significant difference between the groups.
(ns, non-significant; * *p* < 0.05; ** *p* ≤ 0.01; *** *p* ≤ 0.001).

© 2017 by the authors; licensee MDPI, Basel, Switzerland. This article is an open access article distributed under the terms and conditions of the Creative Commons by Attribution (CC-BY) license (http://creativecommons.org/licenses/by/4.0/).
